# Conducting high-quality and reliable acoustic analysis: A tutorial focused on training research assistants

**DOI:** 10.1121/10.0025536

**Published:** 2024-04-17

**Authors:** Elizabeth Heller Murray

**Affiliations:** Department of Communication Sciences and Disorders, Temple University, Philadelphia, Pennsylvania 19122, USA

## Abstract

Open science practices have led to an increase in available speech datasets for researchers interested in acoustic analysis. Accurate evaluation of these databases frequently requires manual or semi-automated analysis. The time-intensive nature of these analyses makes them ideally suited for research assistants in laboratories focused on speech and voice production. However, the completion of high-quality, consistent, and reliable analyses requires clear rules and guidelines for all research assistants to follow. This tutorial will provide information on training and mentoring research assistants to complete these analyses, covering areas including RA training, ongoing data analysis monitoring, and documentation needed for reliable and re-creatable findings.

## I. INTRODUCTION

There has been a growing emphasis on using open science practices in research in recent years. Open science practices encourage researchers to make their articles and data openly accessible to their peers, fostering collaboration, transparency, accountability, and reproducibility in the scientific community. In fact, new National Institutes of Health (NIH) regulations require the inclusion of a “Data Management and Sharing Plan” in all federally funded grant applications. Moreover, creating and maintaining these sharing plans can be included in the budget, allowing researchers to allocate funds to complete this important endeavor. Within the specific field of speech-language pathology, there has been additional emphasis on the importance of open science and data sharing in recent years. Groups such as the OpenCSD community have formed from interested scientists and clinicians to support and educate the greater communication sciences and disorders (CSD) community. Their website^1^ contains a wealth of resources and support for those interested in learning more about open science. The *Journal of Speech, Language, and Hearing Research* recently published a forum on open science practices in CSDs, edited by Guest Editor Dr. Rachel Teodore. This issue includes tutorials on preregistration,^2^ a discussion on the importance of including data that can be accessed for future meta-analysis,^3^ and a tutorial discussing reproducibility in studies with only a few participants (small-N).^4^ Additionally, papers were included that examined current open science practices in CSD,^5^ the effect that open access has on metrics of impact,^6^ and the importance of including marginalized populations that are historically excluded from research in open science work,^7^ as well as other important topics in this area.^8–11^

This growth in open science practices will considerably impact the sharing of speech samples from individuals with and without communication disorders. There are a multitude of repositories of audio files available for the interested researcher, with funding for the creation of these repositories provided by the NIH, National Science Foundation, as well as other foundations or internal grants. Some of the larger databases with speakers of American English are outlined below. The repository TalkBank, started by Dr. Brian MacWhinny, provides extensive access to researchers interested in speech and language.^12^ Since its inception, researchers from around the world have added audio files, video files, and often associated transcripts for areas including child language,^13^ aphasia,^14^ fluency,^15^ and many more categories that can be found on their website. Additional details can be found on the TalkBank website,^16^ with the resources reviewed in an article by MacWhinney.^17^ Two recent additions to the TalkBank platform compiled recordings from multiple studies and unpublished works to create a corpus of children with and without speech disorders. One corpus has recordings of children completing the Goldman Fristoe Test of Articulation (PERCEPT-GFTA),^18^ while the other corpus is focused on rhotic production (PERCEPT-R).^19,20^ Outside of the TalkBank platform, databases such as the Speech Exemplar and Evaluation Database (SEED)^21^ were created to share high-quality speech samples from children and adults both with and without speech disorders across the lifespan. This database was designed to provide clinicians and researchers with exemplars of typical and disordered speech.^22^ The Arizona Child Acoustic Database Repository^23^ has speech samples from children without speech disorders recorded longitudinally at 3-month intervals. During the recording period, some children were identified as having suspected speech sound disorders, and these children are specified in the participant descriptions.^24^ The Perceptual Voice Qualities Database^25^ contains voice recordings from adults with and without voice disorders and expert clinician ratings on dysphonia.^26^ Researchers have begun publishing work successfully using these resources,^11,27–30^ and as these databases expand and new ones emerge, this list will continue to grow.

Researchers interested in performing acoustic analysis can take full advantage of these available speech samples. Researchers will no longer be limited to collecting recordings from participants who can travel to their research laboratory, allowing for the examination of larger datasets that will result in more generalizable findings. There are excellent resources available for individuals interested in conducting high-quality acoustic analysis, including tutorials on voice and speech analysis,^31–34^ articles that discuss considerations for speech analysis in disordered populations,^35–37^ and articles that discuss the importance of individualizing analysis settings and having a clear understanding of the speech signal for formant analysis.^38,39^ When contemplating using one or more databases, researchers must consider whether the recording quality will allow accurate acoustic analysis. This will be especially important if the researcher is looking across datasets, as different repositories may have significant differences in audio quality based factors, such as the original recording environment and microphone-to-mouth distance. Voice and vocal quality measures can be particularly susceptible to noise interference in audio samples with low signal-to-noise ratios (SNR). Deliyski and colleagues examined multiple software programs and determined that measures of fundamental frequency and perturbation were best (e.g., minimal measurement error) at 42 dB SNR, with acceptable levels continuing to be present down to 30 dB SNR. Below 30 dB SNR, the acoustic noise interfered too much with the measurements to provide valid data.^40^ Additional examination of vocal measures of fundamental frequency and cepstral peak prominence are relatively robust across recording environments or microphones, whereas harmonics-to-noise ratio, shimmer, and jitter are more sensitive to these factors.^41^ Examination of acoustic analysis of speech measures has shown that automatic formant measurements can also be impacted by background noise or recording devices; however, this can be improved with some manual user input.^38,39,42^ Thus, while automatic analysis of voice and speech is efficient, it is advisable to employ manual or semi-automated analysis methods when working with open source data recorded by others.

Manual and semi-automated analyses require clear rules and guidelines; otherwise, there is the opportunity for significant measurement errors. The purpose of this tutorial is to provide some examples and recommendations for conducting these time-intensive acoustic analyses that are often more suited for data accessed from different databases. Interest in performing the more time-intensive manual acoustic analysis is typically found in research laboratories. Moreover, these laboratories are the ideal place to encourage undergraduate and master's students to become interested in research and for doctoral students and postdoctoral fellows to hone their skills. However, mentoring students on this type of analysis can feel stressful without safeguards in place. Therefore, this tutorial will provide information on training and mentoring research assistants (RAs) to complete high-quality and reliable analyses. I have drawn examples from my own laboratory^43^ as well as reaching out to colleagues for added input from their work (see Acknowledgments); open source scripts and examples highlighted by colleagues are indicated with links in the text below. Information associated with this article will be available through my laboratory Open Science Framework (OSF) page.^44^ Furthermore, this OSF page will be linked to the Speech Data Consortium, a group started by Dr. Marisha Speights Atkins, Dr. Meg Cychoza, Dr. Yolanda Holt, and Dr. Thea Knowles, that houses a growing collection of open access to protocols for speech data collection, analysis, and repositories of recordings.^45^

## II. TRAINING OF RAs

An essential first step to having multiple RAs accurately and consistently complete manual or semi-automated acoustic analysis is a comprehensive training protocol. This training protocol should (1) ensure the RAs understand the analysis, (2) have clear instructions on completing the analysis, and (3) include an element of practice or hands-on work before the RA is cleared to move onto the real data analysis phase.

### A. Understanding the analysis

**FIG. 1.**
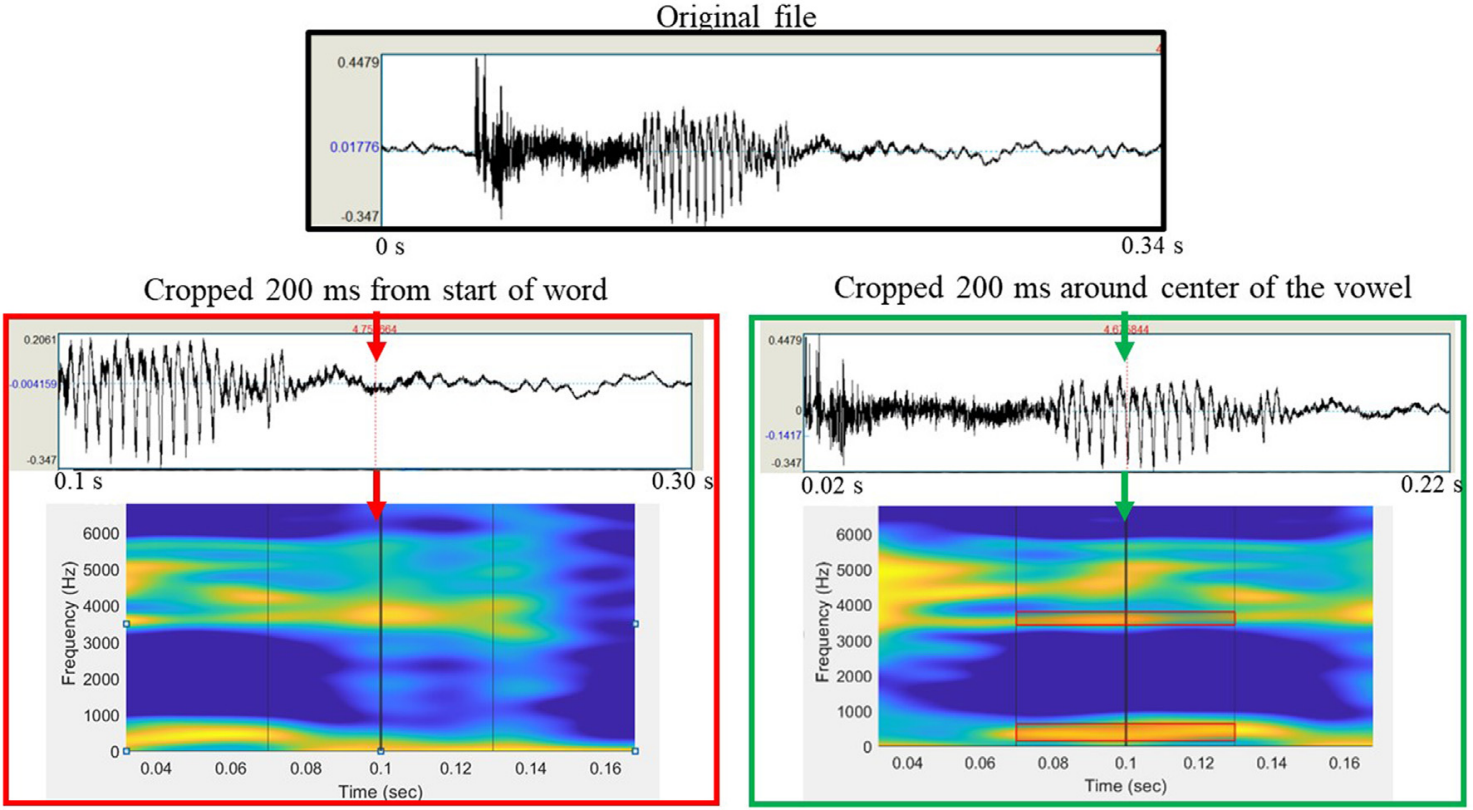
(Color online) RAs must understand the analysis purpose to ensure accuracy. In this example, the RA was instructed to crop the word “peak” for later vowel analysis. Two potential outcomes are shown: (1) the left panel (red box) shows an example of cropping from the beginning of the word, and (2) the right panel (green box) shows an example of cropping around the midpoint. In the example in the red box, later analysis of the vowel would not be possible. In contrast, in the example in the green box, the cropping was correctly completed, and thus would allow later analysis at the vowel midpoint.

Although it may be tempting to focus only on the steps the RAs will take in conducting the analysis, all RAs must clearly understand the analysis goal, regardless of the complexity of the task. Figure 1 shows an example of the potential negative ramifications if the RA does not understand the ultimate goal of the analysis. In this task, an RA was instructed to crop the vowel portion of the word “peak,” ensuring the cropped file saved is at least 200 ms long. At a later time, a different RA will need identify the vowel's first and second formants (i.e., the peak resonant frequencies that characterize a vowel and are formed based on the shape of the vocal tract during speech) from this cropped file. If the purpose of cropping the file was unclear to the RA, it is possible they could crop a 200 ms portion that starts at the beginning of the word. By the time this cropped file reached downstream in the analysis, evaluation of the midpoint of the file would not provide accurate information on the vowel formants (Fig. 1, left). However, if the RA was clear on the instructions and purpose of the analysis, they would first find the center of the vowel and crop a segment that spans 100 ms on each side of the midpoint. This will allow the formants to be accurately analyzed from the center portion of the vowel (Fig. 1, right). Thus, accurate and consistent analysis requires all RAs to understand the purpose of the analysis, not just receive instructions on the task they were individually assigned.

### B. Clear instructions and reference material

To maintain consistency among all RAs, a detailed and comprehensive instructional manual for each type of analysis is needed. Importantly, if this analysis could accurately be replicated by solely pressing a button without any user input, we would all conduct an automatic analysis! At a minimum, the instructional manual should include a clear set of rules on identifying the speech segment of interest and its associated features [see example of criteria outlined for voice onset time (VOT) analyses in Knowles *et al.* (2021)].^46^ The best practice is to have one manual that everyone can access, removing any versioning issues that may add confusion and inconsistencies to the analysis. These manuals can take many forms, depending on what suits the laboratory. For example, they can cover a single analysis type (e.g., all formant analysis), can cover all analysis types in a particular software (e.g., all analysis in praat), can include study-specific information for multiple studies (e.g., formant analysis for XY and YZ studies), or may be a very specific document with the analysis tailored to a single study (e.g., formant analysis on XY study). Today, most labs have online spaces where they can store and share manuals and instructions (e.g., an internal server or password-protected lab website, lab wikis, or collaborative platforms such as Markdown files in GitHub, Microsoft Teams or OneDrive, and Google Drive).

**FIG. 2.**
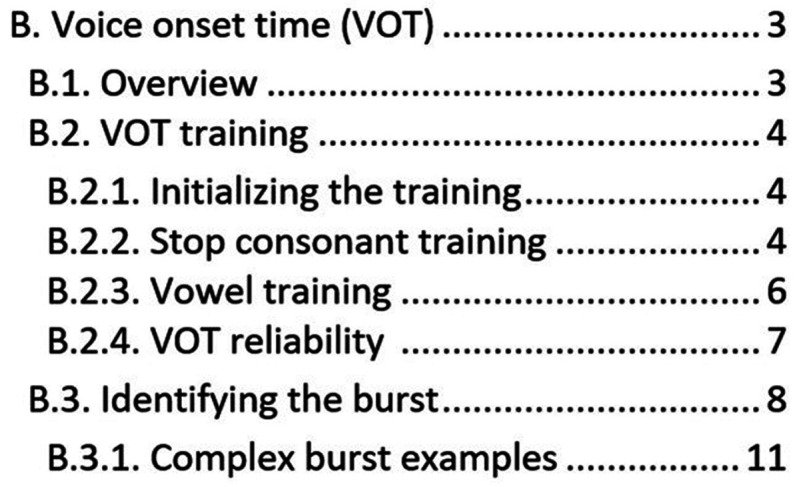
A portion of the table of contents from a voice onset time analysis manual used in Dr. Heller Murray's laboratory.

Figure 2 is a portion of the table of contents from my laboratory from our VOT manual. VOT is a temporal measurement of speech that can be calculated from the waveform. It represents the time between the burst of energy caused by release of the oral construction from a stop consonant and the start of the periodic vocalic signal (e.g., time between the release of the “p” and the start of the “a” in the word “pa”) Unless there is a major change to the analysis flow, most of this manual will remain consistent. If there are changes to the analysis, the best practice is to document the date and reason for these changes in the manual. This makes the manual a “living document” that keeps a record of all changes and outlines, in detail, the current protocols used for analysis. It is always important that the senior researcher can go back and recreate any previous analysis accurately. The manual is also a good place to keep track of difficult or unusual productions so future RAs can benefit from any decisions made on unusual productions. After the senior researcher decides on a difficult case, the RA can update the “complex examples” section of the manual with a picture of the speech sample, the question they posed, and the answer and explanation provided by the senior researcher. Videos are also a useful way to supplement the written manual. Videos can be a casual recording of a training session where a senior researcher is teaching a new RA the analysis or discussing the general concepts related to the analysis. Alternatively, videos can be made specifically for the training protocol and used to show the RAs a clean run-through of the analysis. These videos are often a good place to show complex examples and walk through how the senior researcher made their decisions. Similar to written manuals, videos can be study-specific, analysis-specific, or a mix. It would be helpful to have videos linked or highlighted in a training document if available.

### C. Hands-on practice

**FIG. 3.**
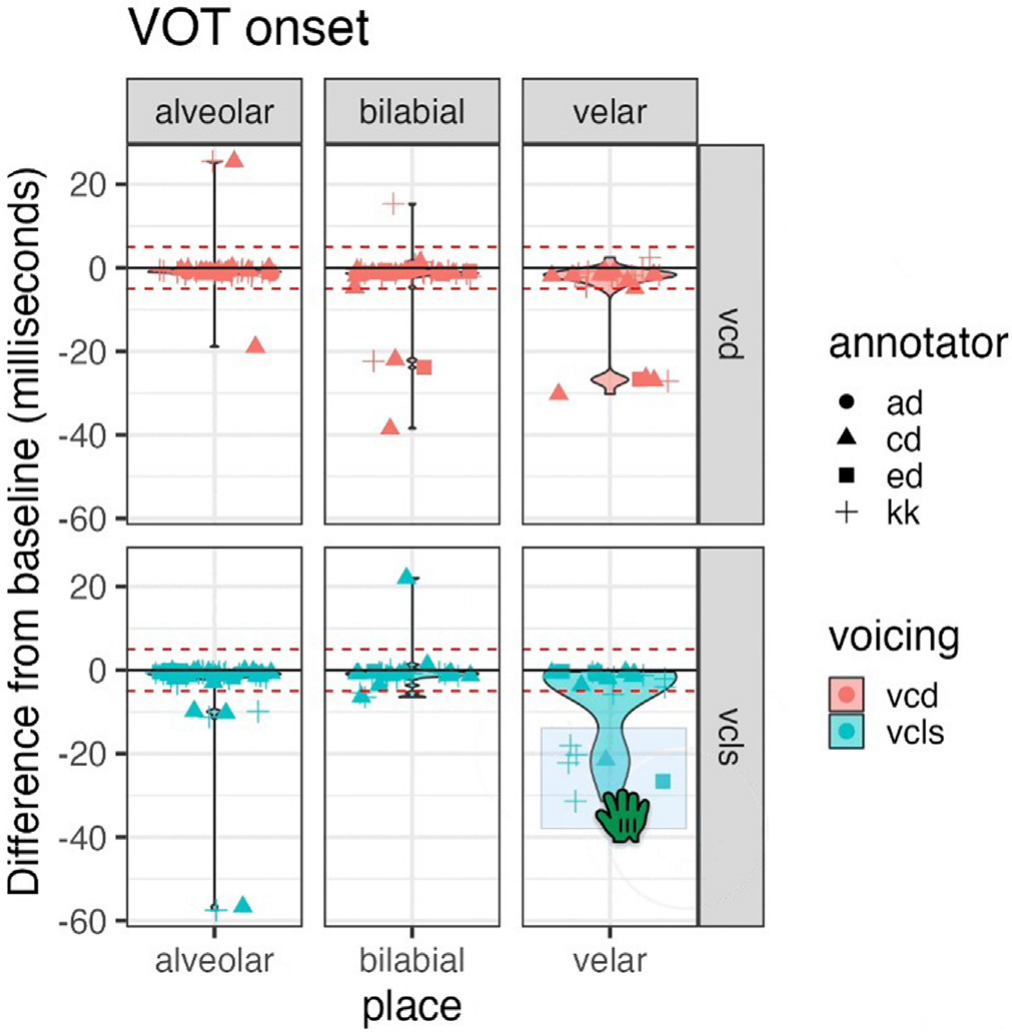
(Color online) Example of an output from an r script reproduced with permission from Dr. Thea Knowles. This script (see Ref. 48) allows the responses from multiple RAs to be compiled and visualized; individual data points of concern can then be selected and reviewed in praat.

When a new RA starts learning an analysis protocol, it is necessary to have a standard set of practice trials for them to go through. For example, the RA could complete a set of annotation tasks, meet to compare annotations, and talk about difficult boundary decisions. Alternatively, RAs may view sets with common errors and have to identify and fix them correctly to show a full understanding of the entire analysis process. It is important this practice include typical examples as well as difficult examples. If the analysis tasks are simpler, having new RAs do these practice trials and talk through them together may be sufficient. However, for more complex analyses, it is beneficial to have RAs try a larger number of trials independently and compare their final responses with a gold standard response completed earlier by the senior researcher. Additionally, by having one set of “correct” values that all future RAs are compared to, researchers can avoid misunderstandings from a training “telephone” situation (e.g., RA1 trains RA2 with incorrect information). These comparisons can provide a quantitative value to examine, such as a correlation or an absolute difference in values. For example, this lab [see example, from Dr. Cara Stepp (Ref. 47)] specifies that RAs must have a Pearson's correlation value of 0.93 or higher with a gold standard rating before they can move past the training stage to actual analysis. For other analysis, it may be more appropriate to look at the absolute difference between one rater and another (e.g., millisecond difference in time between RA and gold standard rating). Alternatively, the senior researcher can create scripts that compile the responses from multiple RAs, allowing them to select points of concern and review them individually [see example from Dr. Knowles (Ref. 48) in Fig. 3].

## III. MONITORING DATA ANALYSIS

### A. Importance of continued check-ins, retraining, and recalibrations

Once an RA has passed all the training protocols, it is tempting to let them just continue on with the analysis without further monitoring. However, everyone benefits from frequent check-ins and an understanding that not all analyses will be straightforward. Some labs like to start lab meetings every week with tricky examples, providing dedicated time to go through all the troublesome cases as a group. Other options are to have a group chat on the lab's preferred platform (e.g., Teams, Slack, etc) where RAs share screenshots and questions about tricky cases. This encourages a natural flow of conversation, promoting a culture of sharing information and asking for help. These chats should include the senior researcher, who can provide a final decision on how to complete the analysis. Once a decision is made, it is also helpful to add that to the manual (e.g., “complex burst examples” heading in Fig. 2), so future RAs can learn from these discussions even if they were not part of the original question. Furthermore, as many RAs are undergraduate students, senior researchers might want to consider smaller retrainings or check-ins after longer breaks to keep all RAs on track.

### B. Clear workflow and established roles

**FIG. 4.**
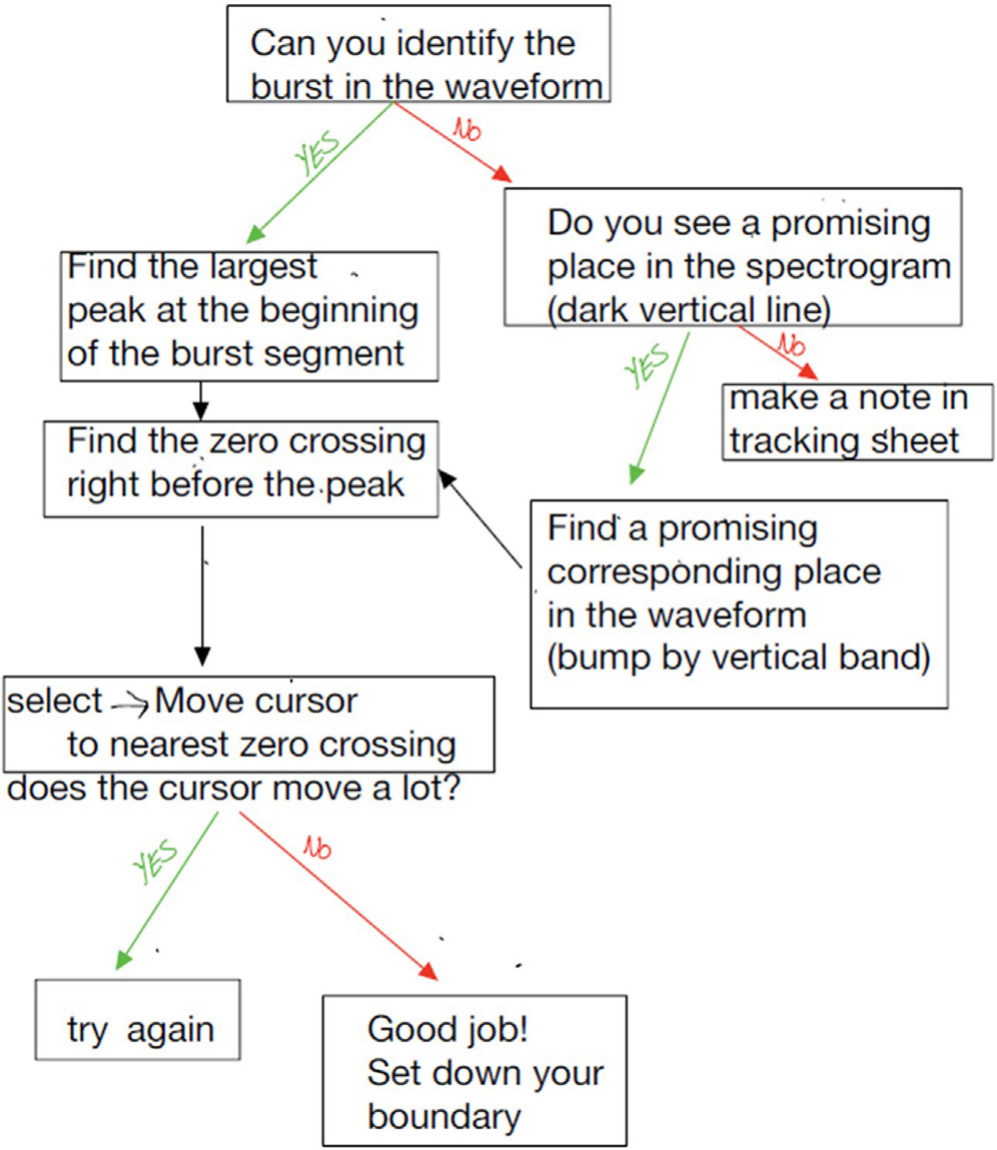
(Color online) Example of workflow outlined in the manual for voice onset time analysis used in Dr. Heller Murray's laboratory. This chart outlines the analysis process and the rules for decision-making. Green arrows show a “yes” path and red arrows show the “no” path for each response.

A clear workflow is essential for any manual or semi-automated analysis. The manual also has a clear workflow for determining the burst's location, including a path for easy and complex examples (see example from our lab's VOT manual in Fig. 4). The workflow documentation should clearly outline each individual's roles and provide guidance on which individual has decision-making capabilities in the analysis. It is important to set clear rules and roles about who can make those decisions and when they can be made. For example, the senior researcher may feel comfortable with an RA skipping a clearly mispronounced file or one that has multiple people talking in the background. Alternatively, an RA may be required to complete all samples they are assigned, but they are also required to write clear notes if there is a case they are less confident about. This can be followed by a secondary look by the senior researcher to confirm the decision was appropriate or make any necessary changes. Ideally, the senior researcher would also independently repeat a certain percentage of the tasks (often 20% is used) to evaluate inter-rater reliability between the RAs and the gold standard values determined by the senior researcher. There is a subjective element to manual analysis; thus, complex or complicated decisions are best left to a single person. This is especially important when examining the speech of individuals with communication disorders, as their speech production is frequently less clear.

### C. Automate the workflow wherever possible

Although the acoustic analysis itself is manual or semi-automated, there are often places in the workflow of this analysis that can be automated. For example, for our lab's VOT analysis in praat, the RAs first find the burst in the raw signal and then identify the vocal onset in a filtered version of the signal. To support this, the praat script used for analysis will first only open the raw signal and prompt the RA to identify all the bursts. Then, after all the bursts are selected, the praat script will open the filtered version for the RA to select the beginning of the vowel. Thus, this ensures that all RAs find those two features the same way. Scripts written in software such as praat, matlab, or python can also be used for a variety of helpful functions, such as establishing the settings before the start of the analysis, saving findings in a particular folder, opening specific productions for review, or a multitude of other functions. These scripts can be extremely useful when multiple RAs are completing the analysis, as they allow the RA to focus all their attention on the complex analysis. By taking care of the things that can be automated, the RAs can focus on the details of the manual analysis.

## IV. DOCUMENTATION DURING ANALYSIS

### A. Tracking settings

In all types of acoustic analyses (manual, semi-automated, automated), the settings selected by the user can have a drastic impact on the outcome. It should be clearly indicated in the manual if the RA is not permitted to edit any settings set by the senior researcher. Ideally, these settings should be saved and exported automatically whenever possible for later review. For example, they could be an automatic log file saved every time an analysis runs that indicates the settings. Some researchers have created their own tailored software (e.g., Ref. 49) to allow the RAs to view files, make decisions on whether the settings fit with the given participant, and make minimal changes if necessary. These decisions and changes are all saved for later review and documentation. Alternatively, when an RA starts an analysis, they can add their name, date, and settings to a running log file. Having these settings documented clearly and accurately is essential for reproducibility of the results.

### B. Tracking who completed the analysis

**FIG. 5.**
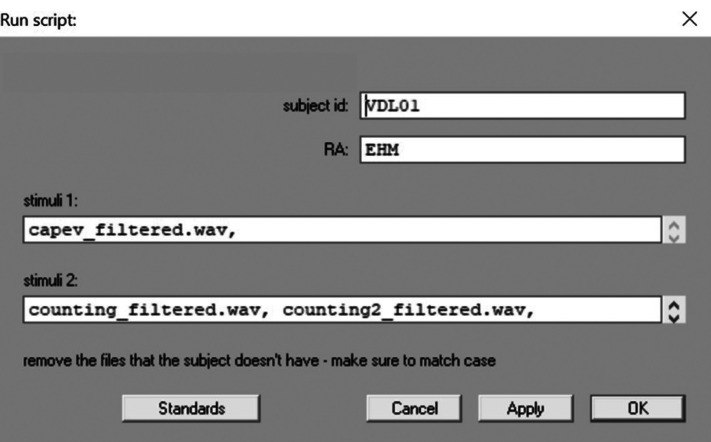
Example of an intake form that opens with a praat script for analysis. This allows automatic documentation of the RA completing the analysis and the sound files to analyze. Once an RA has completed a sample for analysis, the praat script automatically saves the TextGrid with the RA's initials appended (e.g., filename_EHM.textgrid).

When putting together a manuscript or checking reliability, the senior researcher may need to evaluate the analysis from a particular RA or document how many different RAs completed the analysis. This may involve a running an Excel sheet or Google document sheet with the RA names, date, and analysis step completed, or a list of all the subjects that need analysis with a space for the RAs to put their initials when analysis is completed for a given subject. Some labs use more formal management software to assign and track progress on projects, such as asana, slack, or trello. Alternatively, this tracking can be integrated directly into the workflow with additional automation. Figure 5 shows the intake form on a praat script that opens speech samples for the RA to review and place boundary markers in a TextGrid. Once an RA has completed a sample for analysis, the praat script automatically saves the TextGrid with the RA's initials appended (e.g., filename_EHM.textgrid). Therefore, this allows the data to be automatically saved with a clear indication of who completed the analysis.

### C. Notes during analysis

**FIG. 6.**
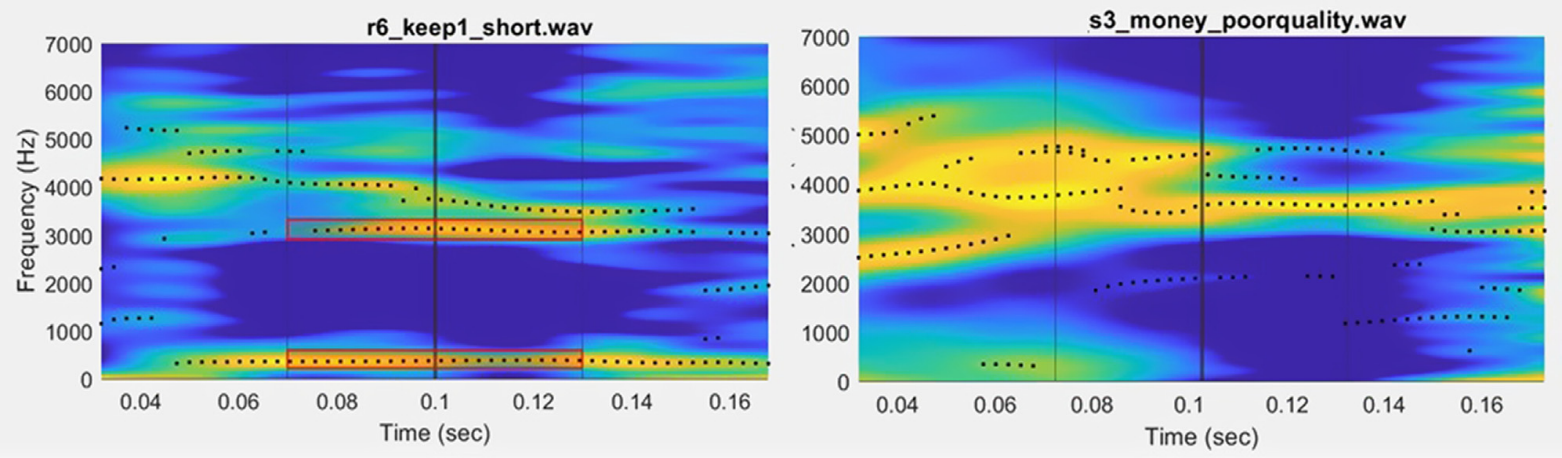
(Color online) Example of output from a formant analysis protocol. The file's title provides information on what the initial RA noted during the initial vowel cropping. This information is helpful for the RA completing the formant analysis at the next stage.

As the RA goes through the analysis, they may notice things that need further review. It would not be practical to stop the analysis to speak to the senior researcher at every point; therefore, having clear ways to document what was noticed is important. This may involve having items built into a script that will automatically move the trial to a new folder that will all be reviewed by the senior researcher.^50^ Alternatively, there may be a running list the RAs can add to for speech samples in which the analysis needs to be reviewed by the senior researcher. When the analysis requires different steps completed by multiple RAs (or even the same RA completing different steps at different times), it is important that information noted early on is not lost in later steps. This may include adding information to a running list that the subsequent RA can check whenever they begin a new subject for analysis. Other times, saving the relevant information directly into the file name may be helpful. Our lab does not shy away from long file names: the more details, the less likely something will get lost in the shuffle! Figure 6 shows an example of how this is helpful in a formant analysis protocol used in my laboratory. In this protocol, the first RA's job is to crop the vowels for analysis. During this process, this first RA noted that the vowel was shorter than expected. That did not mean the speech sample was unusable, but it is important to consider this during the next analysis stage. When the first RA saved the file, they append it with “_short.” The next stage in this protocol opens the file in matlab and uses a formant detection algorithm optimized for children's speech.^51^ The title of the figure shows the notes from the first RA, allowing the second RA completing the formant analysis to have that information readily available. In Fig. 6, the left example is an instance where the information (“_short”) noted during the initial vowel cropping did not impact formant detection, and the subsequent RA was able to highlight the first and second formants (red boxes), whereas, the right example in Fig. 6 shows a file that is likely unusable for formant detection. By having the note “poor quality,” the RA doing formant detection is aware that this speech sample is less than ideal and thus is not surprised when they cannot easily identify the formants.

## V. REVIEWING VALUES BEFORE FINALIZING RESULTS

**FIG. 7.**
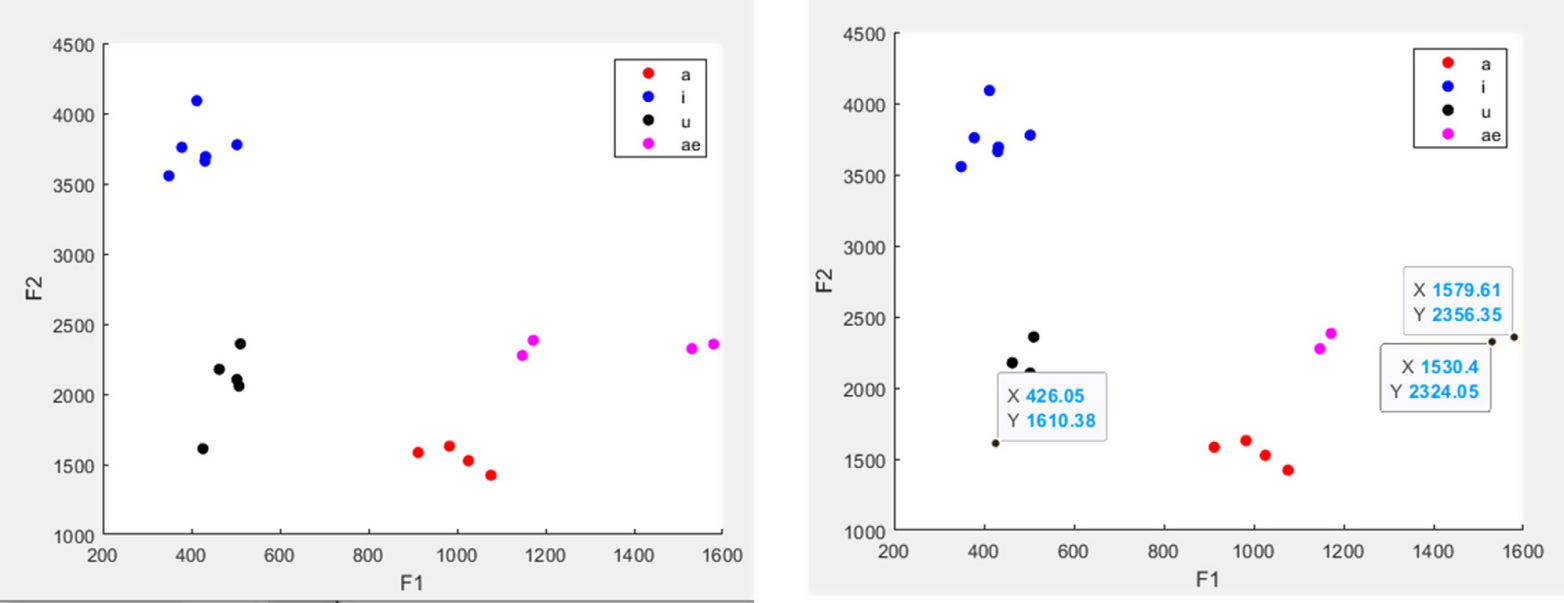
(Color online) Example output from a formant review script (see Ref. 44) used in Dr. Heller Murray's laboratory. This allows examination of formant values in F1-F2 space and selection of individual points for additional review.

The last important step before compiling and analyzing the results is to examine the numerical and/or visual outcomes and ensure they are reasonable. This is important regardless of whether the analysis is automatic or manual. For example, Benway and colleagues discuss in one study that the first step of their review process is to evaluate all the values to ensure they were plausible (e.g., a first formant [F1] value of 5000 Hz is very implausible), which could lead to a manual re-analysis.^52^ Visually evaluating the numerical outputs either per individual or as a larger group can also help outliers stick out. The script written by Dr. Knowles (Fig. 3), shows how individual data points in VOT values can be selected for further analysis. Similarly, Fig. 7 shows a figure output from a formant review script used in my laboratory. In this example, the first (F1) and second (F2) formant values extracted from a single child are shown in the left-hand figure. This script allows the senior researcher to select specific points to review. After selection, the spectrogram with the location of the F1 and F2 values identified by the RA is shown for review (see red boxes on spectrogram in Fig. 1). The senior researcher can either decide to keep the values, redo the formant analysis, or remove the values from the analysis completely. Alternatively, as this particular analysis can be very complex in our clinical populations of interest, the senior researcher can review each spectrogram to see where the RA identified F1 and F2 and make any necessary changes. Although this can be more time-intensive for the senior researcher, reviewing each production is exponentially quicker than doing the original analysis. Thus, the senior researcher will benefit from having a well-trained RA complete the original analysis and still have the opportunity to review all the results prior to a formal analysis of the entire study.

## VI. CONCLUSION

Research that relies on acoustic analysis can provide a wealth of information on voice and speech production. Although automated analyses are faster, it is often inappropriate for clinical populations or when examining data from different databases. These more complex audio files are best analyzed with manual or semi-automated analysis methods, which can be more time-intensive. Due to the time-intensive nature of manual analysis, they are often completed by RAs in a research laboratory. Thus, researchers interested in this analysis must clearly outline all protocols and training procedures. It is essential that the training of RAs (1) ensure the RAs understand the analysis, (2) have clear instructions on completing the analysis, and (3) include an element of practice or hands-on work before the RA is cleared to move onto the real data analysis phase. Once RAs are trained, ongoing monitoring of data analysis should include (1) continued check-ins, retraining, and recalibrations of RAs if needed, (2) a clear workflow with clearly established roles, and (3) the senior researcher automating part of the workflow whenever possible. Finally, documentation of (1) analysis settings, (2) who completed the analysis, and (3) anything noted during the analysis will be essential for accurately tracking the analysis and recreating the findings if needed at a later time. With careful planning, clear documentation, and transparent and well-documented decision-making, any researcher can complete accurate and reliable acoustic analyses.

## Data Availability

Data sharing is not applicable to this article as no new data were created or analyzed in this study.
